# A Novel *Aeromonas popoffii* Phage AerP_220 Proposed to Be a Member of a New *Tolavirus* Genus in the *Autographiviridae* Family

**DOI:** 10.3390/v14122733

**Published:** 2022-12-07

**Authors:** Vera Morozova, Yuliya Kozlova, Ghadeer Jdeed, Artem Tikunov, Tatyana Ushakova, Alevtina Bardasheva, Elena Zhirakovskaia, Yuliya Poletaeva, Elena Ryabchikova, Nina V. Tikunova

**Affiliations:** 1 Laboratory of Molecular Microbiology, Institute of Chemical Biology and Fundamental Medicine, Siberian Branch of Russian Academy of Sciences, Novosibirsk 630090, Russia; 2 Faculty of Natural Sciences, Novosibirsk State University, Novosibirsk 630090, Russia

**Keywords:** *Autographiviridae*, *Aeromonas popoffii*, bacteriophage, comparative genomics, proteomic analysis, tail fiber, *Tolavirus*

## Abstract

*Aeromonas popoffii* is one of the environmental *Aeromonas* species. A number of factors of virulence have been described for this species and it has been reported as a causative agent of urinary tract infection. The first *A. popoffii* bacteriophage AerP_220 along with its host strain *A. popoffii* CEMTC 4062 were isolated from river water. The phage has a podovirus morphotype, shows a narrow host range and is lytic against the host strain. The AerP_220 genome comprises 45,207 bp and does not contain genes responsible for antibiotic resistance and toxin production. Fifty-nine co-directional putative ORFs were found in the AerP_220 genome. Thirty-three ORFs encoded proteins with predicted functions; the products of 26 ORFs were hypothetical proteins. AerP_220 genome analysis revealed that this phage can be considered a novel species within the *Autographiviridae* family. Comparative genomic and proteomic analysis revealed that AerP_220 along with the *Aeromonas* phage vB_AspA_Tola (OM913599) are members of a new putative *Tolavirus* genus in the family *Autographiviridae*. The *Gajwadongvirus* and proposed *Tolavirus* genera along with Pantoea phage Nufs112 and phage Reminis could form a new *Tolavirinae* subfamily within the *Autographiviridae* family.

## 1. Introduction

The genus *Aeromonas* belongs to the *Aeromonadaceae* family and currently comprises 31 species (https://lpsn.dsmz.de/genus/aeromonas (accessed on 7 March 2022)). These Gram-negative, rod-shaped bacteria are able to produce of virulence determinants, biologically active extracellular enzymes, toxins, and biofilms [1]. *Aeromonas* bacteria are widely distributed in aquatic environments and can also be isolated from non-aquatic habitats, animals, and food, especially “ready-to-eat” seafood meals. Moreover, some *Aeromonas* spp. can grow relatively uninhibited in food during refrigeration in various packaging atmospheres under a broad range of pH and NaCl concentrations. Strains of several *Aeromonas* species have shown the ability to produce spoilage-associated metabolites in various seafood products [2]. Given its high prevalence in seafood and in vegetables, *Aeromonas* bacteria are often called “emerging foodborne pathogens”. Wound infections are the second most common type of human infections associated with *Aeromonas*; they occur upon contact of the wounds with fresh water or soil [3,4,5]. In addition, it has been reported that using *Hirudo medicinalis* after surgery may lead to *Aeromonas veronii* infection, and sometimes it can progress to subcutaneous abscess and sepsis [6]. As well as *A. veronii*, several other *Aeromonas* bacteria are able to cause a wide spectrum of diseases in humans, and the majority of the strains associated with clinical cases are *Aeromonas caviae*, *Aeromonas dhakensis*, and *A. hydrophila* [1,3]. Other species usually linked with fish diseases, such as *Aeromonas salmonicida*, have also been reported in human infections [7]. Treatment of these infections is complicated by the fact that *Aeromonas* species are naturally resistant to penicillins, a number of cephalosporins, and erythromycin. Ciprofloxacin is consistently active against *Aeromonas* strains, but some resistant cases have been reported [8,9,10,11].

*Aeromonas popoffii* was first described in 1998 [12]. Later, a number of potential factors of virulence were described for this species [13], and it has been reported associated with urinary tract inflammation [14]. Taking into account the natural resistance of the genus *Aeromonas*, it is necessary to develop alternative therapies, one of which may be the use of bacteriophages.

More than a hundred *Aeromonas* phages have been isolated and taxonomically characterized; most of their complete genomes are currently available in the GenBank database (https://www.ncbi.nlm.nih.gov/genomes/GenomesGroup.cgi?taxid=10239&host=bacteria, accessed on 15 August 2022). Previous studies have been predominantly focused on lytic phages and their biological activity against *A. hydrophila* and *A. salmonicida* [15,16,17,18,19]; therefore, the majority of studied *Aeromonas* phages (*n* = 73) are specific to strains of these two *Aeromonas* species. The most prevalent taxonomic groups of *Aeromonas* phages are members of the *Straboviridae* and *Autographiviridae* families. Note that among *A. hydrophila* phages, 15 members of *Autographiviridae* and 15 phages with myovirus morphology have been identified (eight *Straboviridae* and seven unclassified *Myoviridae* members). In contrast, *A. salmonicida* phages mostly belong to the *Straboviridae* family (17 phages) and only three *Autographiviridae* phages have been found. The remaining phages include *Aeromonas dhakensis* phage P19 (OM339718), *Aeromonas media* phage phiO18P (NC_009542), two *Aeromonas rivipollensis* phages (NC_054465; NC_054466), two *Aeromonas veronii* phages (OL799327; OL770365), and 14 phages that have *Aeromonas* spp. hosts. Note, three lytic *A. veronii* phages VTCCBPA5 [20], BUCT695 (OL799327), and BUCT696 (OL770365) have been previously studied, but only DNA polymerase gene sequence (MF198408) is available in the GenBank database for VTCCBPA5 phage. No *A. popoffii* phages have been described.

Here we report a novel *A. popoffii* lytic bacteriophage AerP_220, its biological properties, genome, and possible taxonomy.

## 2. Materials and Methods

### 2.1. Bacterial Host Strain Isolation, Culture Conditions and Susceptibility Testing

To isolate the *A. popoffii* strain, tenfold dilutions of water sample from river Inya, Novosibirsk region were prepared in sterile phosphate buffered saline (PBS, 5.84 g of NaCl, 4.72 g of Na_2_HPO_4_, and 2.64 g of NaH_2_PO_4_x2H_2_O per 1 L, pH 7.5) and spread on nutrient agar plates (Microgen, Obolensk, Russia). Plates were incubated for 24 h at 28 °C. Grown individual colonies were passaged for three times under the same conditions.

*A. popoffii* was identified by sequencing a 1308 b.p. fragment of the 16S rRNA gene using primers 8F 5′-AGRGTTTGATCCTGGCTCA-3′ and 1350R 5′-GACGGGCGGTGTGTACAAG-3′ as described earlier [21]. All PCR amplicons were gel purified (0.6% SeaKem^®^ GTG-agarose, Lonza, ME, USA) and sequenced (BigDye™Terminator v.3.1 Cycle Sequencing Kit and ABI 3500 Genetic Analyzer, Applied Biosystems, Foster City, CA, USA). The determined nucleotide sequence of 16S rRNA was compared with corresponding nucleotide sequences extracted from the NCBI GenBank database.

The obtained *A. popoffii* strain was grown in nutrient broth (NB; Condalab, Madrid, Spain) for 18 h at various temperatures ranging from 10 °C to 37 °C (10, 16, 20, 25, 28, 31 and 37 °C). In each case, the culture was screened by measurements of OD_600_ every three hours. Three independent experiments were performed for each temperature.

Susceptibility to seven antibiotics was determined using a disk diffusion test according to the guidelines of EUCAST 10.0 (https://eucast.org, accessed on 14 January 2022). Aztreonam (30 mkg), ampicillin (10 mkg), ceftazidime (10 mkg), cefepime (30 mkg), levofloxacin (5 mkg), cyprofloxacin (5 mkg), and erythromycin (15 mkg) were examined. Disks with antibiotics (OXOID, Altrincham, UK) were applied to the lawns of the investigated culture on Mueller–Hinton agar (OXOID, Altrincham, UK).

### 2.2. Phage Isolation and Propagation

To select bacteriophages, 10 mL of water sample that was previously used for *A. popoffii* isolation was sterilized by filtration through a 0.22-mkm filter (Millipore, Guyancourt, France). The filtrate was screened for bacteriophages by spotting 10 mkl aliquots onto a fresh layer of *A. popoffii* CEMTC 4062 in the top agar, containing 0.8% bacteriological agar (OXOID, Altrincham, UK). The plates were incubated overnight at 28 °C and each plaque was suspended in sterile PBS to extract phage particles. Tenfold dilution of obtained phage suspensions were spotted on the fresh layer of *A. popoffii* CEMTC 4062 to obtain single phage plaques for subsequent phage extraction. The cycle of phage dilution and extraction was repeated three times.

Phage AerP_220 was propagated by infecting 100 mL of a culture of *A. popoffii* CEMTC 4062 (OD_600_ = 0.6) grown in NB. Phage particles were added at a multiplicity of infection (MOI, i.e., the ratio of phage to bacterium) of 0.1. The infected culture was incubated at 28 °C for 30 min without shaking and then for several hours with shaking until cell lysis appeared. Phage particles were concentrated from phage lysate by polyethylene glycol 6000 (PEG 6000; AppliChem, Darmstadt, Germany) precipitation as described previously [22].

### 2.3. Phage Plaques and Phage Particles Morphology

The morphology of plaques formed by phage AerP_220 on a layer of sensitive culture *A. popoffii* CEMTC 4062 was determined using the double agar overlay method [23]. Plaques were examined after overnight incubation at 28 °C.

To examine phage particle morphology, a drop of phage AerP_220 suspension (10^9^ pfu/mL) was adsorbed for 1 min on a copper grid covered with formvar film, then the excess liquid was removed, and the grid was contrasted on a drop of 1% uranyl acetate for 5–7 s and examined with a JEM 1400 transmission electron microscope (JEOL, Tokyo, Japan). Digital images were collected using a side-mounted Veleta digital camera (Olympus SIS, Münster, Germany).

### 2.4. Biological Properties of the AerP_220 Phage and Host Range Study

All experiments on the biological properties of phage AerP_220 were performed twice, three times in each repeat. Phage adsorption rate and burst size experiments were carried out as described previously with slight modifications [24,25].

To perform burst size experiments, 10 mL of a mid-exponential-phase culture of *A. popoffii* CEMTC 4062 was pelleted by centrifugation and the pellet was re-suspended in 0.5 mL NB. AerP_220 bacteriophage with an MOI of 0.01 was added to the cell suspension and the mixture was incubated for 5 min at 28 °C for phage adsorption. After that, the cells were pelleted by centrifugation and re-suspended in 10 mL NB. The infected culture was incubated with shaking for 1 h at 28 °C. Culture aliquots were collected every 5 min, and the phage titre was determined.

A lytic activity assay of phage AerP_220 was performed as described previously [26] with our modifications. An exponentially growing culture of *A. popoffii* CEMTC 4062 (10^7^ cfu/mL) was mixed with phage AerP_220 (MOI of 0.001). The mixture was then incubated with shaking at 28 °C. Every 30 min, aliquots were taken, and the appropriate dilutions spread on the nutrient agar plates and incubated overnight at 28 °C. The next day, bacterial colonies were counted. The multistep bacterial killing curve in the phage AerP_220 life cycle was calculated based on the obtained data.

Host range study for the phage AerP_220 was carried out using a spot-assay method [27] on 27 strains of *Aeromonas* spp. from the Collection of Extremophilic Microorganisms and Type Cultures (CEMTC) of the Institute of Chemical Biology and Fundamental Medicine SB RAS, Novosibirsk, Russia.

### 2.5. AerP_220 DNA Purification and Complete Genome Sequencing

Phage DNA was extracted from the phage preparation as described previously [28]. Briefly, phage particles were precipitated using a solution containing 20% PEG 6000 and 2.5 M NaCl, and the pellet was dissolved in STM-buffer (10 mM NaCl; 50 mM Tris-HCl, pH 8.0; 10 mM MgCl_2_). RNase and DNase (Thermo Fisher Scientific, Waltham, MA, USA) were added to the phage preparation to a final concentration 5 mkg/mL, and the mixture was incubated for 1 h at 37 °C. Then, the phage suspension was supplemented with EDTA, proteinase K (Thermo Fisher Scientific, Waltham, MA, USA) and SDS to final concentrations of 20 mM, 100–200 mkg/mL, and 0.5%, respectively, and the mixture was incubated for 3 h at 55 °C. After that, phage DNA was purified by phenol/chloroform extraction and subsequent ethanol precipitation.

A paired-end library of bacteriophage AerP_220 was prepared using the NEBNext Ultra II DNA Library Prep Kit for Illumina (New England BioLab, Ipswich, MA, USA) with NEB Next multiplex oligos for Illumina (New England BioLabs, Ipswich, MA, USA). Sequencing was carried out using the MiSeq Benchtop Sequencer and MiSeq Reagent Kit v.2 (2 × 250 base reads) (Illumina Inc., San Diego, CA, USA). Reads were filtered out by quality with Trimmomatic. The genome was de novo assembled using the SPAdes genome assembler v.3.15.2 (http://cab.spbu.ru/software/spades, accessed on 1 August 2021). Final amount of reads that formed single master contig were 27706 and sequencing depth was estimated to be 132.

To estimate DNA fragments containing 5′- and 3′-end genome sequences, recognition sequences for type II restriction endonucleases were found in the AerP_220 genome using Vector NTI software [29] and phage DNA was digested with endonuclease *Apa*LI (SibEnzyme, Novosibirsk, Russia). The hydrolysed DNA profile was revealed using electrophoresis in 1% agarose gel, and the obtained DNA fragments were compared with the calculated ones. The AerP_220 phage genome sequence was deposited in GenBank with the accession number ON624112.

### 2.6. Genome Analysis

Putative open reading frames (ORFs) were annotated using Rapid Annotation Subsystem Technology (RAST) v.2.0 (https://rast.nmpdr.org, accessed on 16 May 2022), and the obtained annotation was verified manually by checking all of the predicted proteins against the NCBI GenBank protein database (https://www.ncbi.nlm.nih.gov, accessed on 19 May 2022). To compare the products encoded by the predicted ORFs with the sequences deposited in the GenBank database, BLASTX and DELTA-BLAST algorithms were used. InterProScan and HHPred software were used to analyse the predicted ORFs encoding hypothetical proteins and ORFs without homology with the sequences deposited in the GenBank database [30,31]. Screening for t-RNA genes was done using tRNAscan-SE [32].

Search for virulence factors and for antibiotic resistance genes was carried out using the Virulence Factor database (http://www.mgc.ac.cn/VFs, accessed on 31 May 2022), and the Antibiotic Resistance Gene database (https://card.mcmaster.ca/rgi, accessed on 30 May 2022), respectively.

A comparative proteomic phylogenetic analysis was performed using a Viral Proteomic tree server (ViPTree server) (https://www.genome.jp/viptree, accessed on 9 May 2022), comparing phages available in the Virus-Host database (https://www.genome.jp/virushostdb, accessed on 9 May 2022) and NCBI GenBank database. Intergenomic similarity (S_G_) was calculated using the same ViPTree server and Virus Intergenomic Distance Calculator, VIRIDIC (http://rhea.icbm.uni-oldenburg.de/VIRIDIC accessed on 9 May 2022). vConTACT2 was used to perform protein-sharing network analysis by creating viral clusters (VC). To analyse AerP_220, approximately 3550 genomes of prokaryotic viruses were extracted from the NCBI Refseq database. Markov cluster (MCL) was chosen for protein clustering, and pairs of closely related genomes with a similarity score of ≥1 were grouped into viral clusters (VCs) created by clustering with overlapping neighborhood expansion (ClusterONE). The created network was visualized by Cytoscape 3.9.1 program [33]. CoreGenes 5.0 software (https://coregenes.ngrok.io, accessed on 10 June 2022) was used for pangenome analysis.

Proksee software (https://proksee.ca, access date 3 June 2022) was used for comparative analysis of the AerP_220 genome and genomes of the related phages.

### 2.7. Phylogenetic Analysis of Phage Proteins

The BLASTP program was used to search for the corresponding protein sequences (https://blast.ncbi.nlm.nih.gov/, accessed on 20 June 2022). The selected amino acid sequences were aligned using the ClustalW algorithm. Phylogenetic trees were constructed using the maximum likelihood (ML) method based on the JTT matrix-based LG model in MEGA 7.0 with 1000 bootstrap replicates [34].

## 3. Results and Discussion

### 3.1. Bacterial Host Isolation and Susceptibility Testing

The *Aeromonas popoffii* CEMTC 4062 strain was isolated from a water sample taken from Inya River, Novosibirsk region, Russia. The Inya River is a right tributary of the big Siberian Ob River. The sample was collected in a sterile tube in July 2020 when the water temperature was 26 °C and the air temperature was 31 °C. The isolated strain was identified as *A. popoffii* by sequencing of the 16S rRNA gene, and the sequence was deposited in the GenBank database under accession number ON479602. The strain *A. popoffii* CEMTC 4062 was mesophilic and was able to grow at temperature of 25 °C and above with optimal growth temperature of 28 °C.

Based on the data on *Aeromonas* spp. natural antimicrobial resistance [8,9,10], susceptibility of *A. popoffii* CEMTC 4062 to a number of antibiotics was determined. *A. popoffii* CEMTC 4062 was found to be resistant to beta-lactams (penicillin, ampicillin, ceftazidime, cefepime) and erythromycin; it was sensitive to fluoroquinolones (levofloxacin, ciprofloxacin), and aztreonam. This strain was deposited in CEMTC.

### 3.2. AerP_220 Plaques and Phage Morphology

Bacteriophage AerP_220 was obtained from the same water sample as the host strain *A. popoffii* CEMTC 4062. Prior to phage isolation, an aliquot of the sample was sterilized by filtration. Phage AerP_220 formed large clear plagues on a bacterial lawn of *A. popoffii* CEMTC 4062. Plaques had a diameter of approximately 1–2 mm and were surrounded by a translucent halo, probably associated with the activity of phage lytic enzymes (Figure S1).

**Figure 1:**
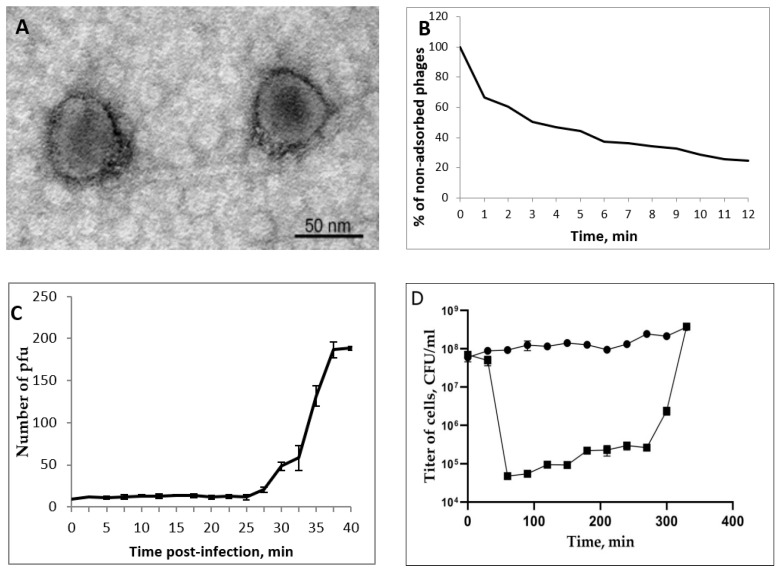
Properties of the AerP_220 phage. Electron micrograph of AerP_220 phage particles negatively stained with 1% uranyl acetate (**A**). AerP_220 phage adsorption curve (**B**). Burst size and latent period (**C**). Multistep bacterial killing curve (**D**); black diamonds indicate *A. popofii*, gray squares indicate *A. popofii* infected with AerP_220.

Electron microscopy of phage AerP_220 particles revealed icosahedral heads with a diameter of 50–55 nm connected toa short tail of approximately 10–12 nm in length (Figure 1A). The morphology and size of phage particles corresponded to the podovirus morphotype [35].

### 3.3. AerP_220 Biological Properties and Host Range

More than 75% of the AerP_220 phage particles attached to host cells after 12 min in phage adsorption experiments (Figure 1B). A one-step growth curve for phage AerP_220 revealed a latent period duration of about 30 min with a burst size of ~20 phage particles per infected cell (Figure 1C). The multistep bacterial killing curve of the phage life cycle revealed lytic properties of the phage AerP_220 (Figure 1D). The amount of living bacteria decreased by three orders in the hour following phage infection, after which it started to increase slowly. Thus, the obtained data demonstrated high lytic activity of phage AerP_220 against the sensitive strain *A. popoffii* CEMTC 4062.

A host range assay was carried out using 27 *Aeromonas* spp. strains, which were isolated mostly from river and lake water samples (Table S1) and deposited in CEMTC. Phage AerP_220 was able to infect only *A. popofii* CEMTC 4062 from all tested *Aeromonas* species. Notably, it did not infect *A. veronii* CEMTC 4064 that was isolated from the same water sample as the *A. popofii* CEMTC 4062 (Table S1). This corresponds to a general tendency of narrow host range for phages of podovirus morphotypes. AerP_220 was found to be strain-specific, since two other *A. popoffii* strains CEMTC 1430 and CEMTC 3381 from the collection were not susceptible to AerP_220. However, a limited number of *A. popofii* strains were tested, and it cannot be excluded that AerP_220 could infect several strains if more *Aeromonas* strains were screened.

Phages of podovirus morphotype, which infect Gram-negative bacteria, usually need polysaccharide moieties for irreversible adsorption on the bacterial surface. However, limited data are available about *A. salmonicida* and *A. hydrophyla* phage receptors [36,37,38] and no information was found for other *Aeromonas* species.

### 3.4. Genome Characteristics

**Figure 2:**
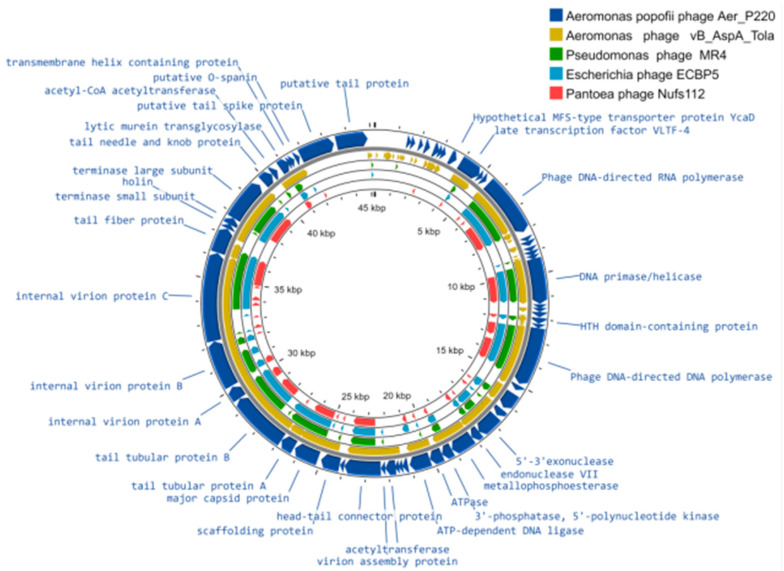
*Aeromonas* phage AerP_220 genome map visualized using the Proksee software. Open reading frames (ORFs) of the AerP_220 genome are denoted with blue in the outer circle. Only proteins with predicted functions are signed on the map. The arrows in the inner circles represent actual protein-coding sequences of significantly similar regions. The TBLASTX algorithm was used for comparative genome alignment of the AerP_220, *Aeromonas* phage vB_AspA_Tola (OM913599), *Pseudomonas* phage MR4 (MT104467), *Escherichia* phage ECBP5 (KJ749827), and *Pantoea* phage Nufs112 (OK570185) genomes.

The size of the assembled AerP_220 genome was determined as 45,207 bp. Fifty-nine predicted ORFs were found in the AerP_220 genome (Supplementary Materials, Data S1). Products of 33 ORFs were found to be proteins with known functions; 26 amino acid sequences were determined as hypothetical proteins (Data S1). Genes encoding virulence factors, antibiotic resistance genes, and tRNA genes were not found in the genome of AerP_220. All predicted ORFs are co-directional and grouped into three functional clusters. The precise determination of functional clusters is difficult, because the AerP_220 genome contains some potential ORFs without homology with known sequences. The DNA-dependent RNA polymerase gene is located between the early and the DNA metabolism gene clusters (Figure 2). It is typical for members of the *Autographiviridae* family to have the gene that encodes single subunit RNA polymerase. The first cluster contains approximately eleven early genes and only two of them encode proteins with predicted functions. The rest of the genes are probably expressed at the beginning of infection, which provides metabolic changes in cells necessary for the subsequent development of phage infection. The second cluster comprises more than 20 DNA metabolism genes, including several predicted genes; the DNA ligase gene is located at the downstream end of the cluster. The third cluster of the late genes is associated with the assembly of phage capsids, DNA maturation, and the outburst of mature phage particles (Data S1, Figure 2). Most genes from this cluster (22/26 ORFs) encode proteins with predicted functions and 14 of them are presumably structural genes.

Three different tail fiber proteins were annotated—AerP_220_p48, AerP_220_p58, and AerP_220_p59. The greater part of the ORF48 encoding AerP_220_p48 is similar to the gene encoding the tail fiber adaptor protein Tola_p58 (UOW66401) of another *Aeromonas* phage vB_AspA_Tola described previously [19]. The N-terminal part of the Tola_p58 resembles the gp17 of *Escherichia* T7 phage, which is responsible for connection with the baseplate [39]. AerP_220_p58 was annotated as a tail spike protein. It shows no similarity with tail spikes of other *Aeromonas* phages. The low similarity of AerP_220_p58 (~89% coverage and ~20% identity) was only revealed with tail spikes (APD20249; QZI85319; YP_009600510) of *Acinetobacter* phages AM24 (KY000079), BUCT629 (MZ712044), and WCHABP12 (NC_041924), respectively. Using InterProScan software, pectate lyase domain was found in the AerP_220_p58. Downstream of the ORF58, an additional gene was identified, encoding a putative tail spike protein AerP_220_p59. No similarity with other phage tail proteins was recorded for AerP_220_p59 using Protein BLAST algorithms, and the possible function of this protein was predicted using HHPred software. Like AerP_220_p58, AerP_220_p59 contains polysaccharide-hydrolyzing domain, so both proteins might be responsible for the formation of a halo around the plaques on the host strain culture. We suppose that AerP_220_p58 and AerP_220_p59 connect with the AerP_220 virion through AerP_220_p48, which forms a linkage between these proteins and the phage capsid. Probably, both AerP_220_p58 and AerP_220_p59 provide binding to bacterial receptor(s).

### 3.5. Comparative Analysis of the AerP_220 Genome with Other Genomes

The AerP_220 genome was compared with other phage genomes available in the NCBI GenBank Database using BLASTN. The highest similarity was with the genome of another *Aeromonas* phage vB_AspA_Tola (OM913599) [19] with sequence identity of 73.62% and coverage of 55%. Some similarity was found between the AerP_220 genome fragments and those of *Pseudomonas* phage MR4 (MT104467), *Escherichia* phage ECBP5 (KJ749827), *Pantoea* phage Nufs112 (OK570185); however, coverage was < 5%. Comparison of the AerP_220 phage genome and genomes of the listed phages using the Proksee software revealed clear gene synteny (Figure 2).

**Figure 3:**
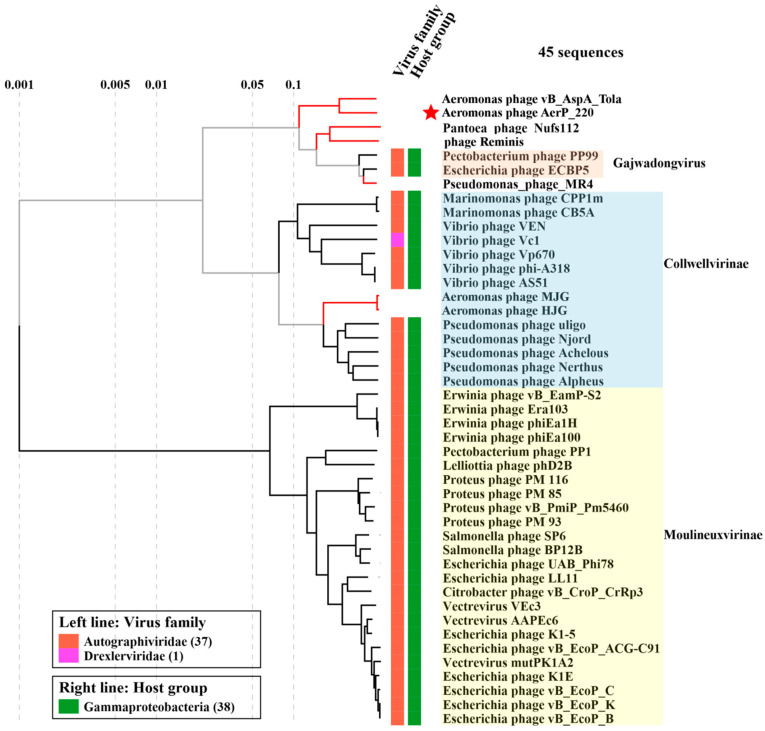
Comparative proteomic phylogenetic analysis of phage AerP_220 and similar phages performed using VipTree. The investigated phage AerP_220 is marked with a red asterisk. Red phylogenetic branches mark phage sequences which were not found in Virus-Host Database. These genomes were downloaded from the NCBI GenBank database and added to the analysis manually.

Comparative proteomic phylogenetic analysis was performed using ViPTree software. It was shown that the AerP_220 and vB_AspA_Tola genomes formed a separate branch within a cluster containing genomes of *Pantoea* phage Nufs112 (OK570185), bacteriophage Reminis (MN478376), *Pectobacterium* phage PP99 (NC047802), *Escherichia* phage ECBP5 (KJ749827), and *Pseudomonas* phage MR4 (MT104467) (Figure 3). Two phages from the cluster, *Escherichia* phage ECBP5 and *Pectobacterium* phage PP99 were previously classified as members of the *Gajwadongvirus* genus, *Autographiviridae* family (ICTV Master Species List 2021.v1; https://talk.ictvonline.org, accessed on 9 May 2022). The described cluster was closer to the *Colwellvirinae* and *Moulineuxvirinae* than to other subfamilies in the *Autographiviridae* family (Figure 3). Other *Aeromonas* phages from the *Autographiviridae* family (Table S2) including MJG (MK455769) and, HJG (MK455770) [40] had very low identity with AerP_220 and did not cluster with AerP_220 and vB_AspA_Tola phages (Figure 3).

**Figure 4:**
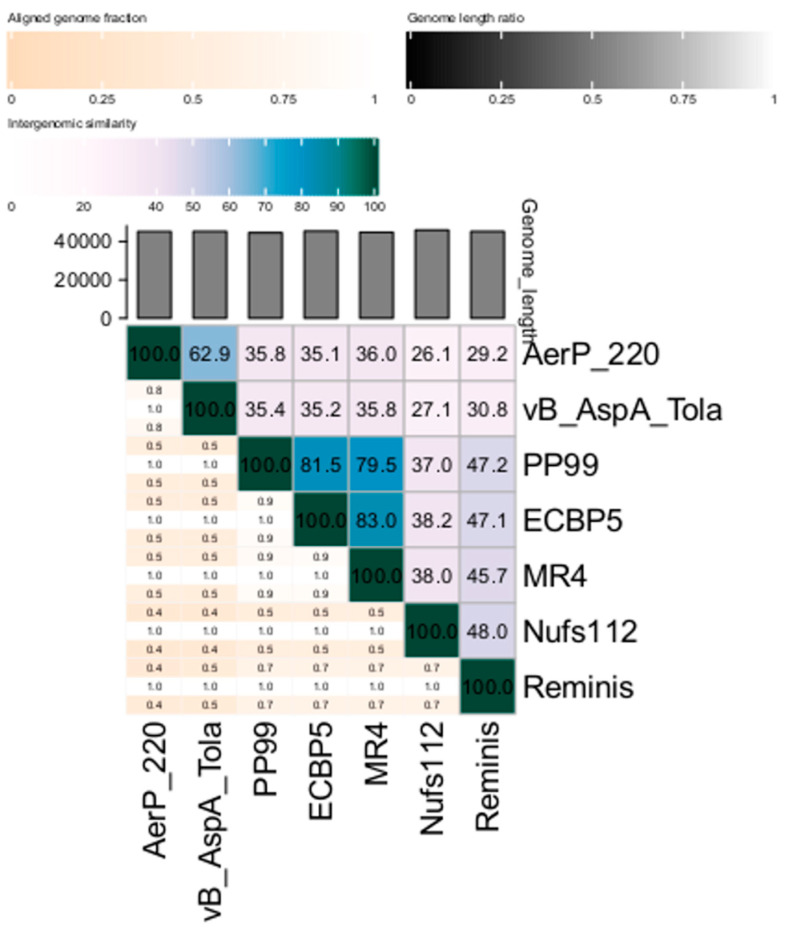
Matrix of intergenomic similarities determined using VIRIDIC for *Aeromonas* phage AerP_220, *Aeromonas* phage vB_AspA_Tola (OM913599), *Pectobacterium* phage PP99 (NC047802), *Escherichia* phage ECBP5 (KJ749827), *Pseudomonas* phage MR4 (MT104467), *Pantoea* phage Nufs112 (OK570185), and phage Reminis (MN478376). Color coding scales are represented above the matrix.

To further examine the taxonomy of the AerP_220 phage, matrix of intergenomic similarities of the AerP_220 genome with six most similar phage genomes (*Aeromonas* phage vB_AspA_Tola, *Pectobacterium* phage PP99, *Escherichia* phage ECBP5, *Pseudomonas* phage MR4, *Pantoea* phage Nufs112, and phage Reminis) was calculated using VIRIDIC (Figure 4). Pairwise similarity of genomes in most cases was lower than the cut-off, (70% nucleotide identity of the complete genome length) established by ICTV Bacterial Virus Subcommittee for creating phage genera [41]. The exception was the genomes of three phages, *Pseudomonas* phage MR4, *Escherichia* phage ECBP5, and *Pectobacterium* phage PP99. Identity levels confirmed that these phages belong to the same genus, the *Gajwadongvirus* genus.

Intergenomic similarity of the AerP_220 and vB_AspA_Tola genomes, calculated using VIRIDIC, was close to the demarcation of phage genera but lower than 70% (Figure 4). However, both phages infect *Aeromonas* hosts, and their genomes have significant gene synteny. Moreover, a set of 41 homologous conserved signature genes was revealed in-between AerP_220 and vB_AspA_Tola genomes (Table 1, Data S2).

Based on these facts and proteomic ViPTree analysis, we assume that the investigated phage AerP_220 and *Aeromonas* phage vB_AspA_Tola form a new genus. We suggest naming this new genus *Tolavirus,* after the first member of the proposed genus.

### 3.6. Phylogenetic Analysis of AerP_220 Proteins

Phylogenetic analysis of products encoded by the signature genes (terminase large subunit, DNA directed RNA polymerase, and major capsid protein) was performed using their predicted amino acid sequences. Initial trees for the heuristic search were obtained automatically by applying Neighbor-Join and BioNJ algorithms to a matrix of pairwise distances estimated using a JTT model, and then selecting the topology with superior log likelihood value. Identical sequences were removed from the analysis and all positions containing gaps and missing data were eliminated as well. The final analysis involved the most similar 35 amino acid sequences.

Phylogenetic analysis of terminase large subunit sequences demonstrated that AerP_220 and vB_AspA_Tola protein sequences formed a well-supported group, and this group clustered with corresponding sequences of *Pantoea* phage Nufs112, bacteriophage Reminis, and three members of the *Gajwadongvirus* genus (Figure S2A). The topology of the phylogenetic trees of RNA polymerase and major capsid protein sequences were similar, and the corresponding proteins of the above phages were positioned in a similar way (Figure S2B,C). These phylogenetic trees were in accordance with the results of proteomic ViPTree analysis.

Presumably, phages AerP_220 and vB_AspA_Tola (members of the suggested *Tolavirus* genus), members of the *Gajwadongvirus* genus, along with the unclassified *Pantoea* phage Nufs112 and phage Reminis, form a new putative subfamily within the *Autographiviridae* family. We suggest naming it *Tolavirinae.* To test this hypothesis, the similarity (S_G_) of 46 genomes, included into proteomic phylogenetic analysis, was calculated using VipTree software (Table 2). Similarity in-between phages of the proposed *Tolavirinae* subfamily was comparable to those within the *Colwellvirinae* and *Moulineuxvirinae* subfamilies and was higher than similarity between these subfamilies (Table 2).

### 3.7. Protein-Based Similarity Networks

**Figure 5:**
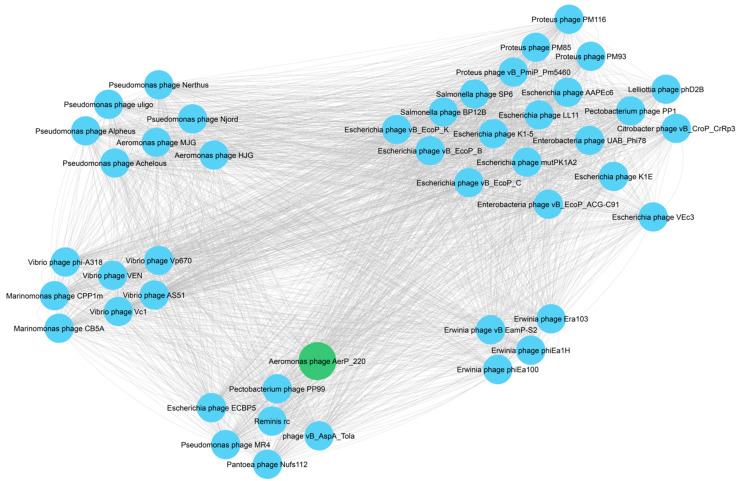
Protein-based similarity network of phage AerP_220 with the most similar phages, vConTACT2 software was used for analysis. The investigated phage AerP_220 is marked with a green circle.

In addition, a protein-based similarity network was constructed for AerP_220 phage along with the most similar phages using vConTACT2 software. Five clusters were revealed; two of them contained phages from the *Colwellvirinae* subfamily, two clusters included members of the *Moulineuxvirinae* subfamily, and one cluster was formed by the AerP_220, vB_AspA_Tola, PP99, ECBP5, MR4, Nufs112, and Reminis phages (Figure 5).

The obtained data confirmed that AerP_220 phage and six of the above-mentioned phages could be members of a new putative *Tolavirinae* subfamily.

**Figure 6:**
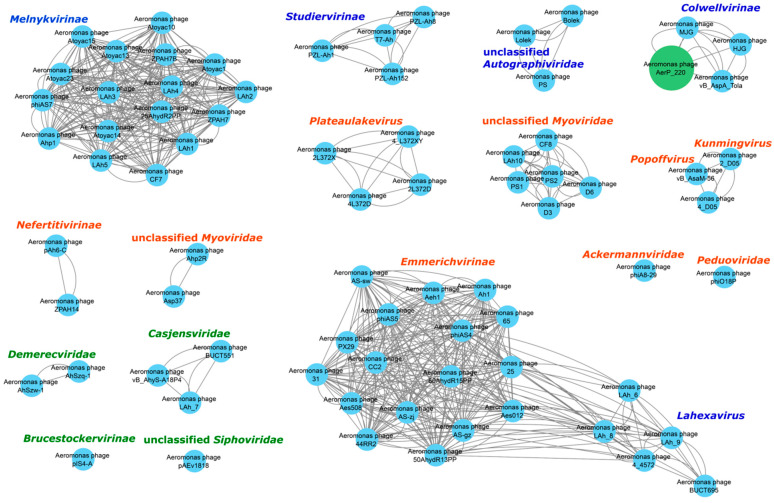
Protein-based similarity network of phage AerP_220 with *Aeromonas* phages, available in the GenBank database (accessed on 15 June 2022). vConTACT2 software was used for analysis. The investigated phage AerP_220 is marked with a green circle. Phage taxonomy groups marked with blue have podovirus morphotype, marked with red have myovirus morphotype, and marked with green, the siphovirus morphotype.

All *Aeromonas* phages available in the GenBank database belong to different taxonomy groups within the Caudoviricetes class. *Aeromonas* myophage genomics and biology have been described previously in comprehensive reviews [17,20]. Recently, a number of new *Aeromonas* phages with podovirus morphotype were described and their genomes appeared in the NCBI GenBank database [19,38,40,42,43,44]. To evaluate the relations between the studied AerP_220 phage and other *Aeromonas* phages, vConTACT2 analysis was performed for all *Aeromonas* phage genomes available in the GenBank database (Table S2, Figure 6). Most of the available *Aeromonas* phages are myophages. A total of 16 independent clusters (groups of phages or individual phages) were identified (Table S2, Figure 6). Of them, four clusters contained phages with podovirus morphotype; seven and four clusters were formed by phages with myovirus and siphovirus morphotypes, respectively. In addition, one unexpected heterogeneous cluster consisted of two subclusters with myophages and podophages: members of the *Straboviridae* family (myovirus morphotype) and *Lahexavirus* genus (podovirus morphotype), the separately standing genus in the last release of phage taxonomy (Figure 6).

Among 33 *Aeromonas* phages with podovirus capsid morphotype, 28 were members of the *Autographiviridae* family and formed four clusters (Figure 6). Two clusters consisted of members of the *Melnykvirinae* (*n* = 17) and *Studiervirinae* (*n* = 4) subfamilies. *Aeromonas* phages PS, Bolek, and Lolek formed the third cluster. One more cluster contained *Aeromonas* phages HJG and MJG, which presumably belong to the *Colwellvirinae* subfamily, and AerP_220 and vB_AspA_Tola, members of the proposed *Tolavirinae* subfamily (Figure 6).

The fact that many *Aeromonas* phages form independent groups, with the exception of myophages from the *Straboviridae* family and podophages from the *Melnykvirinae* subfamily, indicates that despite infecting the same bacterial genus, these phages may exhibit considerable genomic diversity. One possible reason for genetic diversity of *Aeromonas* podovirus phages is the narrow host range inherent to most of them. This peculiarity leads to divergence of genomes in phages that infect only one or two bacterial strains. The obtained data are in good agreement with the previous results of the global analysis of members of the Autographiviridae family [19].

## 4. Conclusions

In this study, the first *A. popoffii* AerP_220 bacteriophage was isolated from the same sample of river water as the host strain, which makes them a true host-pathogen pair existing in this natural niche. AerP_220 demonstrated a virulent lifestyle with a short adsorption and latent period, as well as the ability to reduce the host population by three orders in the hour following phage infection. This phage was found to have a narrow host range. However, it is a lytic phage without pathogenic genes in the genome. Genes that encode exopolysaccharide-degrading enzymatic protein domains were highlighted in silico, and clear plaques with a halo zone suggested that they were active during phage life cycle.

Comparative analysis of the AerP_220 genome with other available genomes revealed that this phage can be considered as a novel species within the *Autographiviridae* family because the nucleotide sequence similarity of AerP_220 to most similar phages differs substantially by more than 5% (criterion established by ICTV Bacterial Virus Subcommittee). To our best knowledge, AerP_220 is the first *Aeromonas* phage of the *Autographiviridae* family that has two genes encoding different predicted tail spike proteins along with the gene encoding the gp17-like tail protein. Nucleotide and proteomic phylogenetic analyses showed that *Aeromonas* phage vB_AspA_Tola is the most similar to the AerP_220 phage. Although the nucleotide identity of AerP_220 and vB_AspA_Tola is somewhat lower than the boundary established by ICTV Bacterial Virus Subcommittee for creating phage genera [41], we propose to combine these phages into one genus named *Tolavirus*. In addition, two monophyletic genera, the proposed *Tolavirus* and *Gajwadongvirus*, along with several unclassified non-*Aeromonas* phages, may form a new *Tolavirinae* subfamily within the *Autographiviridae* family. Notably, only 28 *Aeromonas* phages from the *Autographiviridae* family have been described, and further isolation and characterization of new phages from the family may modify the proposed taxonomy.

## Figures and Tables

**Table 1:** Pangenome analysis of the AerP_220 and a set of most similar phage genomes, performed using CoreGenes 5.0 software.

Number of Homologous Genes	Proposed *Tolavirinae* Subfamily, *n* = 7		*Colwellvirinae*, *n* = 14	*Moulineuxvirinae*, *n* = 25
	Proposed *Tolavirus* Genus, *n* = 2	*Gajwadongvirus* Genus, *n* = 3		
Between members of the genera	41	41		
Between members of the proposed subfamily	26		18	17
Between members of the subfamilies	13			

**Table 2:** Similarity of phage genomes included into phylogenetic analysis.

	Proposed *Tolavirinae* Subfamily, *n* = 7	*Colwellvirinae* Subfamily, *n* = 14	*Moulineuxvirinae* Subfamily, *n* = 25
proposed *Tolavirinae*	0.26 < S_G_ < 0.85	0.16 < S_G_ < 0.21	0.14 < S_G_ < 0.2
*Colwellvirinae*	0.16 < S_G_ < 0.21	0.28 < S_G_ < 0.97	0.14 < S_G_ < 0.22
*Moulineuxvirinae*	0.14 < S_G_ < 0.2	0.14 < S_G_ < 0.22	0.3 < S_G_ < 0.76

## Data Availability

Not applicable.

## Supplementary Materials

The following are available online at: https://www.mdpi.com/article/10.3390/v14122733/s1. Figure S1: Phage AerP_220 plagues on a bacterial lawn of *Aeromonas popoffii* CEMTC 4062. Figure S2: Phylogenetic analysis of three essential proteins of the investigated *Aeromonas* phage AerP_220 along with the most similar proteins: A, terminase large subunit; B, capsid protein; C, DNA-dependent RNA polymerase. GenBank identifiers for the protein sequences are shown in parentheses. The Maximum Likelihood method was used to construct the trees, 1000 bootstrap replications were applied. Statistical support above 70% is shown at the nodes. The sequence IDs of proteins of the investigated *Aeromonas phage* AerP_220 are marked with red circles. The appropriate proteins of *Aeromonas* phage phiAS7 were used as outgroup. Table S1: *Aeromonas* spp. strains screened in host range assay. Table S2: A list of *Aeromonas* phages available in the NCBI GenBank database. Data S1: *Aeromonas* phage AerP_220 genome annotation. Data S2: Pangenome analysis of AerP_220 genome and similar phage genomes.
