# Supplementary material for: A Novel Aeromonas popoffii Phage AerP_220 Proposed to Be a Member of a New Tolavirus Genus in the Autographiviridae Family
Source: Viruses. 2022 Dec 7;14(12):2733. doi: 10.3390/v14122733 (PMC9780818; doi:10.3390/v14122733)
Supplement: Supplementary file 1 [file viruses-14-02733-s001.zip › Table S2.pdf]

# Table S2. A list of Aeromonas phages available in the NCBI GenBank database

| No | Bacterial host | Prototype phage | NCBI Genome ID | Family | Subfamily | Genus |
|---|---|---|---|---|---|---|
| 1 | *A. dhakensis* | Aeromonas phage P19 | OM339718 | Straboviridae | Emmerichvirinae | Ceceduovirus |
| 2 | *A. hydrophila* | Aeromonas phage MJG | MK455769 | Autographiviridae | Colwellvirinae | unclassified Uliginvirus |
| 3 | *A. hydrophila* | Aeromonas phage HJG | MK455770 | Autographiviridae | Colwellvirinae | unclassified Uliginvirus |
| 4 | *A. hydrophila* | Aeromonas phage ZPAH7 | MH992513 | Autographiviridae | Melnykvirinae | Aerosvirus |
| 5 | *A. hydrophila* | Aeromonas phage ZPAH7B | MK330684 | Autographiviridae | Melnykvirinae | Aerosvirus |
| 6 | *A. hydrophila* | Aeromonas phage 25AhydR2PP | NC_047966 | Autographiviridae | Melnykvirinae | Aerosvirus |
| 7 | *A. hydrophila* | Aeromonas phage Ahp1 | NC_047866 | Autographiviridae | Melnykvirinae | Ahphunavirus |
| 8 | *A. hydrophila* | Aeromonas phage CF7 | NC_047865 | Autographiviridae | Melnykvirinae | Ahphunavirus |
| 9 | *A. hydrophila* | Aeromonas phage LAh1 | MK838107 | Autographiviridae | Melnykvirinae | unclassified Ahphunavirus |
| 10 | *A. hydrophila* | Aeromonas phage LAh2 | MK838108 | Autographiviridae | Melnykvirinae | unclassified Ahphunavirus |
| 11 | *A. hydrophila* | Aeromonas phage LAh3 | MK838109 | Autographiviridae | Melnykvirinae | unclassified Ahphunavirus |
| 12 | *A. hydrophila* | Aeromonas phage LAh4 | MK838110 | Autographiviridae | Melnykvirinae | unclassified Ahphunavirus |
| 13 | *A. hydrophila* | Aeromonas phage LAh5 | MK838111 | Autographiviridae | Melnykvirinae | unclassified Ahphunavirus |
| 14 | *A. hydrophila* | Aeromonas phage PZL-Ah8 | MZ851152 | Autographiviridae | Studiervirinae | unclassified |
| 15 | *A. hydrophila* | Aeromonas phage PZL-Ah1 | MT681669 | Autographiviridae | Studiervirinae | Teseptimavirus |
| 16 | *A. hydrophila* | Aeromonas phage PZL-Ah152 | MW671054 | Autographiviridae | Studiervirinae | unclassified |
| 17 | *A. hydrophila* | Aeromonas phage LAh_7 | NC_052985 | Casjensviridae | - | Sharonstreetvirus |
| 18 | *A. hydrophila* | Aeromonas phage vB_AhyS-A18P4 | NC_052984 | Casjensviridae | - | Sharonstreetvirus |
| 19 | *A. hydrophila* | Aeromonas phage BUCT551 | NC_052986 | Casjensviridae | - | Sharonstreetvirus |
| 20 | *A. hydrophila* | Aeromonas phage AhMtk13b | OL840901 | Casjensviridae | - | unclassified Sharonstreetvirus |
| 21 | *A. hydrophila* | Aeromonas phage pAh6-C | NC_048774 | Chaseviridae | Nefertitivirinae | Pahsextavirus |
| 22 | *A. hydrophila* | Aeromonas phage pAh6.2TG | MZ336020 | Chaseviridae | Nefertitivirinae | unclassified Pahsextavirus |
| 23 | *A. hydrophila* | Aeromonas phage PVN02 | MT623578 | Chaseviridae | Nefertitivirinae | unclassified Pahsextavirus |
| 24 | *A. hydrophila* | Aeromonas phage PVN03 | MW380983 | Chaseviridae | Nefertitivirinae | unclassified Pahsextavirus |
| 25 | *A. hydrophila* | Aeromonas phage PVN04 | MW380984 | Chaseviridae | Nefertitivirinae | unclassified Pahsextavirus |
| 26 | *A. hydrophila* | Aeromonas phage PVN05 | MW380985 | Chaseviridae | Nefertitivirinae | unclassified Pahsextavirus |
| 27 | *A. hydrophila* | Aeromonas phage ZPAH14 | OM810291 | Chaseviridae | Nefertitivirinae | unclassified Pahsextavirus |
| 28 | *A. hydrophila* | Aeromonas phage AhSzw-1 | NC_047950 | Demerecviridae | - | Shenzhenvirus |
| 29 | *A. hydrophila* | Aeromonas phage AhSzq-1 | NC_047949 | Demerecviridae | - | Shenzhenvirus |

| | | | | | | |
|---|---|---|---|---|---|---|
| 30 | *A. hydrophila* | Aeromonas phage Akh-2 | MK318083 | Demerecviridae | - | unclassified Shenzhenvirus |
| 31 | *A. hydrophila* | Aeromonas phage CC2 | NC_019538 | Straboviridae | Emmerichvirinae | Ceceduovirus |
| 32 | *A. hydrophila* | Aeromonas phage AP1 | MT713136 | Straboviridae | Emmerichvirinae | unclassified Ceceduovirus |
| 33 | *A. hydrophila* | Aeromonas phage ZPAH1 | MW272540 | Straboviridae | Emmerichvirinae | unclassified Ishigurovirus |
| 34 | *A. hydrophila* | Aeromonas phage Aeh1 | NC_005260 | Straboviridae | - | Cinqassovirus |
| 35 | *A. hydrophila* | Aeromonas phage Ah1 | NC_055733 | Straboviridae | - | Cinqassovirus |
| 36 | *A. hydrophila* | Aeromonas phage 50AhydR13PP | NC_055746 | Straboviridae | - | Tulanevirus |
| 37 | *A. hydrophila* | Aeromonas phage AhMtk13a | OL840900 | Straboviridae | - | Tulanevirus |
| 38 | *A. hydrophila* | Aeromonas phage 60AhydR15PP | NC_055747 | Straboviridae | - | Tulanevirus |
| 39 | *A. hydrophila* | Aeromonas phage Ahp2R | KX455876 | unclassified Myoviridae | unclassified | unclassified |
| 40 | *A. hydrophila* | Aeromonas phage 4_4572 | NC_048773 | unclassified | unclassified | Lahexavirus |
| 41 | *A. hydrophila* | Aeromonas phage LAh_6 | NC_048774 | unclassified | unclassified | Lahexavirus |
| 42 | *A. hydrophila* | Aeromonas phage LAh_8 | NC_048775 | unclassified | unclassified | Lahexavirus |
| 43 | *A. hydrophila* | Aeromonas phage LAh9 | NC_048776 | unclassified | unclassified | Lahexavirus |
| 44 | *A. hydrophila* | Aeromonas phage D3 | MN102098 | unclassified Myoviridae | unclassified | unclassified |
| 45 | *A. hydrophila* | Aeromonas phage D6 | MN131137 | unclassified Myoviridae | unclassified | unclassified |
| 46 | *A. hydrophila* | Aeromonas phage LAh10 | MK838116 | unclassified Myoviridae | unclassified | unclassified |
| 47 | *A. hydrophila* | Aeromonas phage CF8 | MK774614 | unclassified Myoviridae | unclassified | unclassified |
| 48 | *A. hydrophila* | Aeromonas phage PS1 | MN032614 | unclassified Myoviridae | unclassified | unclassified |
| 49 | *A. hydrophila* | Aeromonas phage PS2 | MN453779 | unclassified Myoviridae | unclassified | unclassified |
| 50 | *A. hydrophila* | Aeromonas phage 4L372D | NC_048771 | unclassified | unclassified | Plateaulakevirus |
| 51 | *A. hydrophila* | Aeromonas phage 4_L372XY | NC_048772 | unclassified | unclassified | Plateaulakevirus |
| 52 | *A. media* | Aeromonas phage phiO18P | NC_009542 | Peduoviridae | - | Bielevirus |
| 53 | ***A.popoffii*** | **Aeromonas phage AerP_220*** | **ON624112** | **Autographiviridae** | - | - |
| 54 | *A. rivipollensis* | Aeromonas phage 2_D05 | NC_054465 | unclassified | unclassified | Kunmingvirus |
| 55 | *A. rivipollensis* | Aeromonas phage 4_D05 | NC_054466 | unclassified | unclassified | Kunmingvirus |
| 56 | *A. salmonicida* | Aeromonas phage phiAS7 | NC_019528 | Autographiviridae | Melnykvirinae | Aerosvirus |
| 57 | *A. salmonicida* | Aeromonas phage T7-Ah | MT740748 | Autographiviridae | Studiervirinae | unclassified |
| 58 | *A. salmonicida* | Aeromonas phage AS-sw | NC_048674 | Straboviridae | Emmerichvirinae | Ceceduovirus |
| 59 | *A. salmonicida* | Aeromonas phage AS-zj | NC_048673 | Straboviridae | Emmerichvirinae | Ceceduovirus |

| | | | | | | |
|---|---|---|---|---|---|---|
| 60 | *A. salmonicida* | Aeromonas phage AS-szw | MF498773 | Straboviridae | Emmerichvirinae | Ceceduovirus |
| 61 | *A. salmonicida* | Aeromonas phage AS-yj | MF498774 | Straboviridae | Emmerichvirinae | Ceceduovirus |
| 62 | *A. salmonicida* | Aeromonas phage 65 | NC_015251 | Straboviridae | Emmerichvirinae | Ishigurovirus |
| 63 | *A. salmonicida* | Aeromonas phage PX29 | NC_023688 | Straboviridae | - | Angelvirus |
| 64 | *A. salmonicida* | Aeromonas phage 31 | NC_007022 | Straboviridae | - | Biquartavirus |
| 65 | *A. salmonicida* | Aeromonas phage 44RR2.8t | NC_005135 | Straboviridae | - | Biquartavirus |
| 66 | *A. salmonicida* | Aeromonas phage L9-6 | KY290956.1 | Straboviridae | - | Unclassified Biquartavirus |
| 67 | *A. salmonicida* | Aeromonas phage Riv-10 | KY290957 | Straboviridae | - | Unclassified Biquartavirus |
| 68 | *A. salmonicida* | Aeromonas phage SW69-9 | KY290958 | Straboviridae | - | Unclassified Biquartavirus |
| 69 | *A. salmonicida* | Aeromonas phage phiAS5 | NC_014636 | Straboviridae | - | Chrysonvirus |
| 70 | *A. salmonicida* | Aeromonas phage 25 | NC_008208 | Straboviridae | - | Tulanevirus |
| 71 | *A. salmonicida* | Aeromonas phage phiAS4 | NC_014635 | Straboviridae | - | Tulanevirus |
| 72 | *A. salmonicida* | Aeromonas phage AS-gz | NC_042019 | Straboviridae | - | Tulanevirus |
| 73 | *A. salmonicida* | Aeromonas phage vB_AsM_ZHF | MW584871 | Straboviridae | - | Tulanevirus |
| 74 | *A. salmonicida* | Aeromonas phage Aes508 | NC_019543 | Straboviridae | - | Tulanevirus |
| 75 | *A. salmonicida* | Aeromonas phage Asp37 | KY290949 | unclassified Myoviridae | unclassified | unclassified |
| 76 | *A. salmonicida* | Aeromonas phage 51 | KY290953 | unclassified | unclassified | Popoffvirus |
| 77 | *A. salmonicida* | Aeromonas phage vB_AsaM-56 | NC_019527 | unclassified | unclassified | Popoffvirus |
| 78 | *A. salmonicida* | Aeromonas phage PS | MT259468 | Autographiviridae | unclassified | unclassified |
| 79 | *Aeromonas* sp. | Aeromonas phage phiA8-29 | NC_048660 | Ackermannviridae | - | Tedavirus |
| 80 | *Aeromonas* spp. | Aeromonas phage Atoyac1 | MT682386 | Autographiviridae | Melnykvirinae | unclassified |
| 81 | *Aeromonas* spp. | Aeromonas phage Atoyac10 | MT682387 | Autographiviridae | Melnykvirinae | unclassified |
| 82 | *Aeromonas* spp. | Aeromonas phage Atoyac13 | MT682388 | Autographiviridae | Melnykvirinae | unclassified |
| 83 | *Aeromonas* spp. | Aeromonas phage Atoyac14 | MT682389 | Autographiviridae | Melnykvirinae | unclassified |
| 84 | *Aeromonas* spp. | Aeromonas phage Atoyac15 | MT682390 | Autographiviridae | Melnykvirinae | unclassified |
| 85 | *Aeromonas* spp. | Aeromonas phage Atoyac23 | MT682391 | Autographiviridae | Melnykvirinae | unclassified |
| 86 | *Aeromonas* sp. | Aeromonas phage vB_AspA_Tola | OM913599 | Autographiviridae | unclassified | unclassified |
| 87 | *Aeromonas* sp. | Aeromonas phage Bolek | OM913597 | Autographiviridae | unclassified | unclassified |
| 88 | *Aeromonas* sp. | Aeromonas phage Lolek | OM913598 | Autographiviridae | unclassified | unclassified |
| 89 | *Aeromonas* sp. | Aeromonas phage pAEv1812 | OL964754 | Casjensviridae | - | unclassified Sharonstreetvirus |

| | | | | | | |
|---|---|---|---|---|---|---|
| 90 | *Aeromonas* sp. | Aeromonas phage Aes012 | NC_020879 | Straboviridae | - | Tulanevirus |
| 91 | *Aeromonas* sp. | Aeromonas phage pIS4-A | NC_042037 | - | Brucestockervirinae | Roufvirus |
| 92 | *Aeromonas* sp. | Aeromonas phage pAEv1818 | OL964755 | unclassified Siphoviridae | unclassified | unclassified |
| 93 | *A.veronii* | Aeromonas phage BUCT695 | OL799327 | unclassified Podoviridae | unclassified | unclassified |
| 94 | *A. veronii* | Aeromonas phage BUCT696 | OL770365 | Chaseviridae | Nefertitivirinae | unclassified Pahsextavirus |

*the investigated phage AerP_220
