# Supplementary material for: A Novel Aeromonas popoffii Phage AerP_220 Proposed to Be a Member of a New Tolavirus Genus in the Autographiviridae Family
Source: Viruses. 2022 Dec 7;14(12):2733. doi: 10.3390/v14122733 (PMC9780818; doi:10.3390/v14122733)
Supplement: Supplementary file 1 [file viruses-14-02733-s001.zip › Figure S2 revised.pdf]

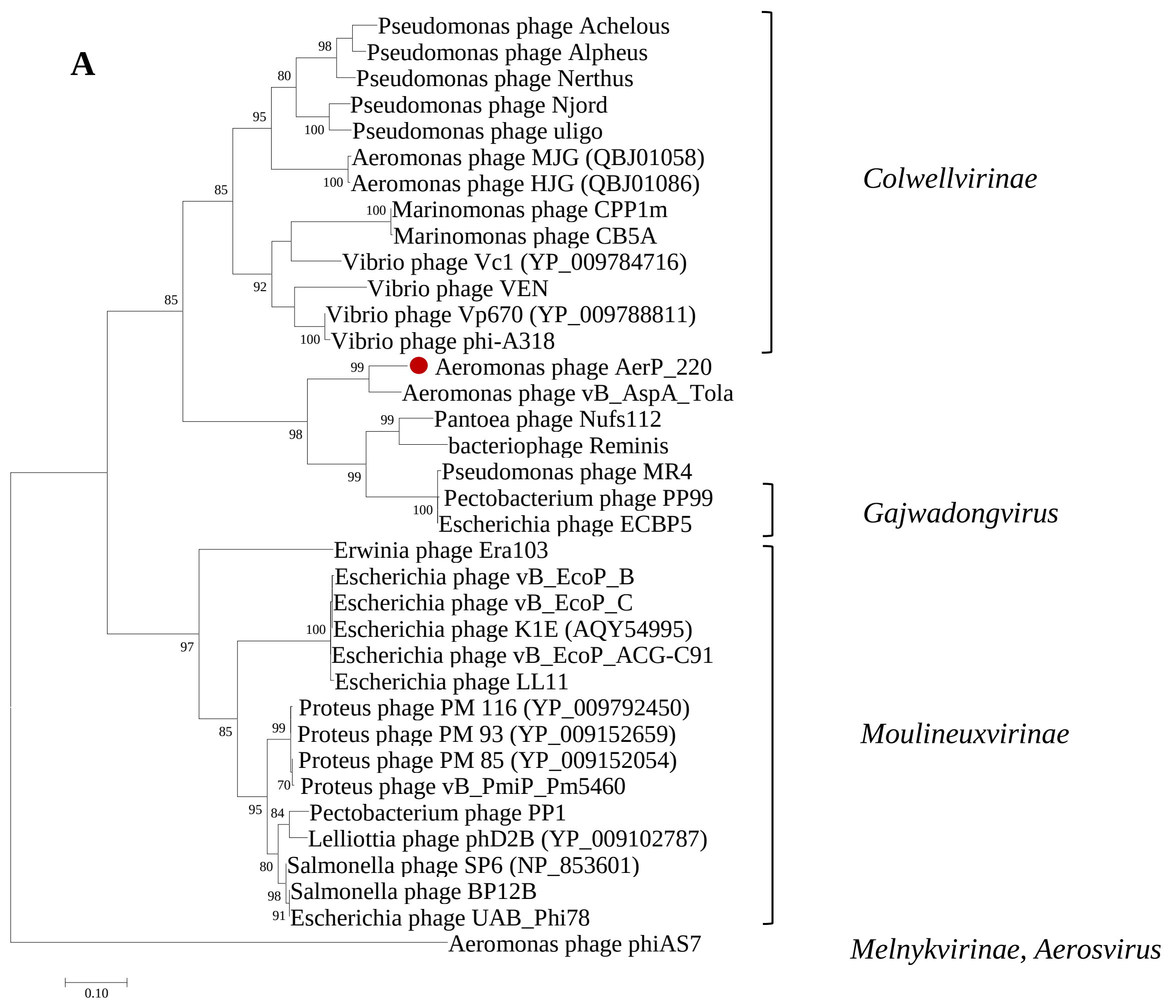
A
Pseudomonas phage Achelous
Pseudomonas phage Alpheus
Pseudomonas phage Nerthus
Pseudomonas phage Njord
Pseudomonas phage uligo
Aeromonas phage MJG (QBJ01058)
Aeromonas phage HJG (QBJ01086)
Marinomonas phage CPP1m
Marinomonas phage CB5A
Vibrio phage Vc1 (YP_009784716)
Vibrio phage VEN
Vibrio phage Vp670 (YP_009788811)
Vibrio phage phi-A318
Aeromonas phage AerP_220
Aeromonas phage vB_AspA_Tola
Pantoea phage Nufs112
bacteriophage Reminis
Pseudomonas phage MR4
Pectobacterium phage PP99
Escherichia phage ECBP5
Erwinia phage Era103
Escherichia phage vB_EcoP_B
Escherichia phage vB_EcoP_C
Escherichia phage K1E (AQY54995)
Escherichia phage vB_EcoP_ACG-C91
Escherichia phage LL11
Proteus phage PM 116 (YP_009792450)
Proteus phage PM 93 (YP_009152659)
Proteus phage PM 85 (YP_009152054)
Proteus phage vB_PmiP_Pm5460
Pectobacterium phage PP1
Lelliottia phage phD2B (YP_009102787)
Salmonella phage SP6 (NP_853601)
Salmonella phage BP12B
Escherichia phage UAB_Phi78
Aeromonas phage phiAS7
Colwellvirinae
Gajwadongvirus
Moulineuxvirinae
Melnykvirinae, Aerosvirus
0.10

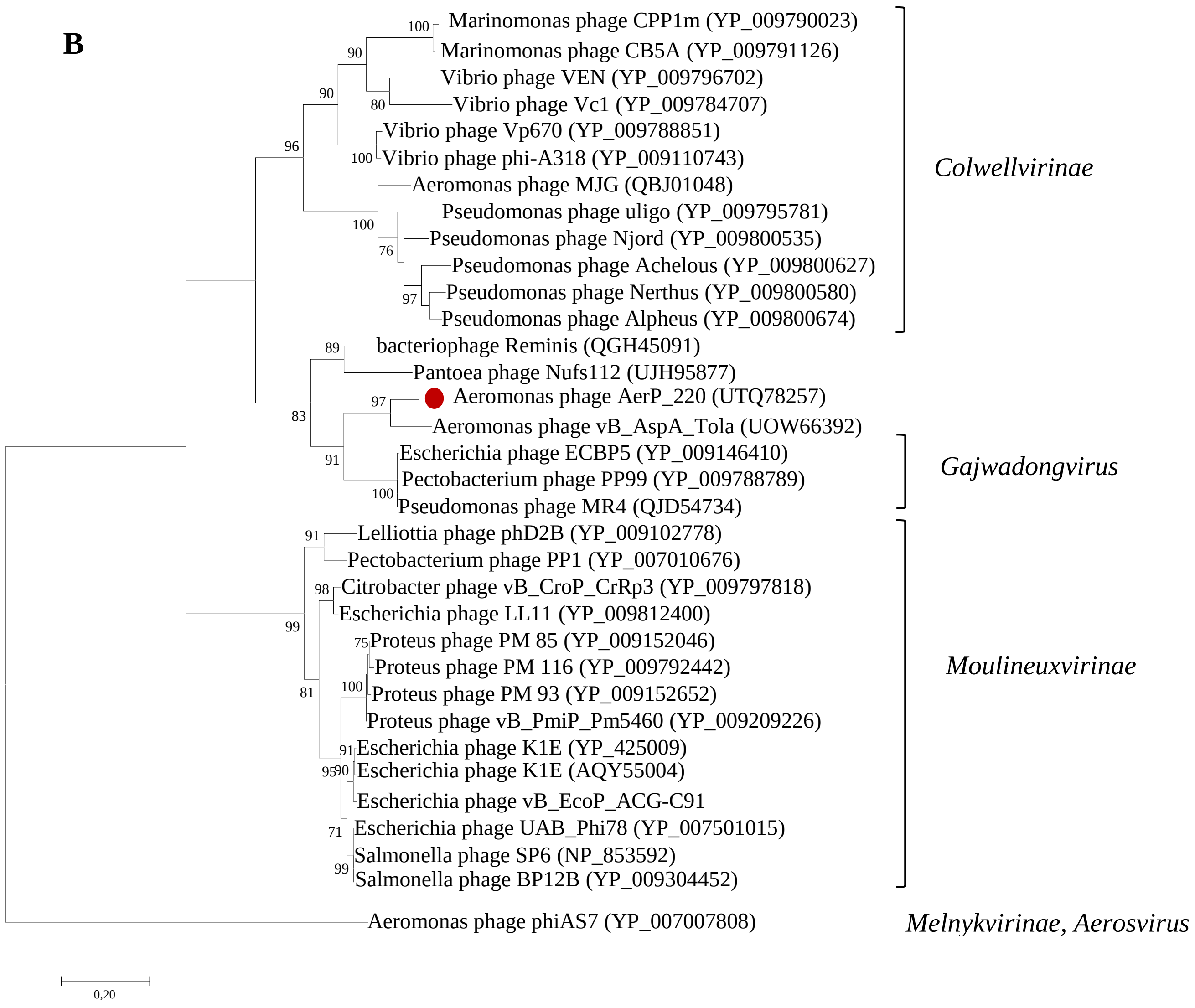
B
Marinomonas phage CPP1m (YP_009790023)
Marinomonas phage CB5A (YP_009791126)
Vibrio phage VEN (YP_009796702)
Vibrio phage Vc1 (YP_009784707)
Vibrio phage Vp670 (YP_009788851)
Vibrio phage phi-A318 (YP_009110743)
Aeromonas phage MJG (QBJ01048)
Pseudomonas phage uligo (YP_009795781)
Pseudomonas phage Njord (YP_009800535)
Pseudomonas phage Achelous (YP_009800627)
Pseudomonas phage Nerthus (YP_009800580)
Pseudomonas phage Alpheus (YP_009800674)
bacteriophage Reminis (QGH45091)
Pantoea phage Nufs112 (UJH95877)
Aeromonas phage AerP_220 (UTQ78257)
Aeromonas phage vB_AspA_Tola (UOW66392)
Escherichia phage ECBP5 (YP_009146410)
Pectobacterium phage PP99 (YP_009788789)
Pseudomonas phage MR4 (QJD54734)
Lelliottia phage phD2B (YP_009102778)
Pectobacterium phage PP1 (YP_007010676)
Citrobacter phage vB_CroP_CrRp3 (YP_009797818)
Escherichia phage LL11 (YP_009812400)
Proteus phage PM 85 (YP_009152046)
Proteus phage PM 116 (YP_009792442)
Proteus phage PM 93 (YP_009152652)
Proteus phage vB_PmiP_Pm5460 (YP_009209226)
Escherichia phage K1E (YP_425009)
Escherichia phage K1E (AQY55004)
Escherichia phage vB_EcoP_ACG-C91
Escherichia phage UAB_Phi78 (YP_007501015)
Salmonella phage SP6 (NP_853592)
Salmonella phage BP12B (YP_009304452)
Aeromonas phage phiAS7 (YP_007007808)
Colwellvirinae
Gajwadongvirus
Moulineuxvirinae
Melnykvirinae, Aerosvirus
100
90
90
80
96
100
100
76
97
89
97
83
91
100
91
98
99
75
100
81
91
95
90
71
99
0,20

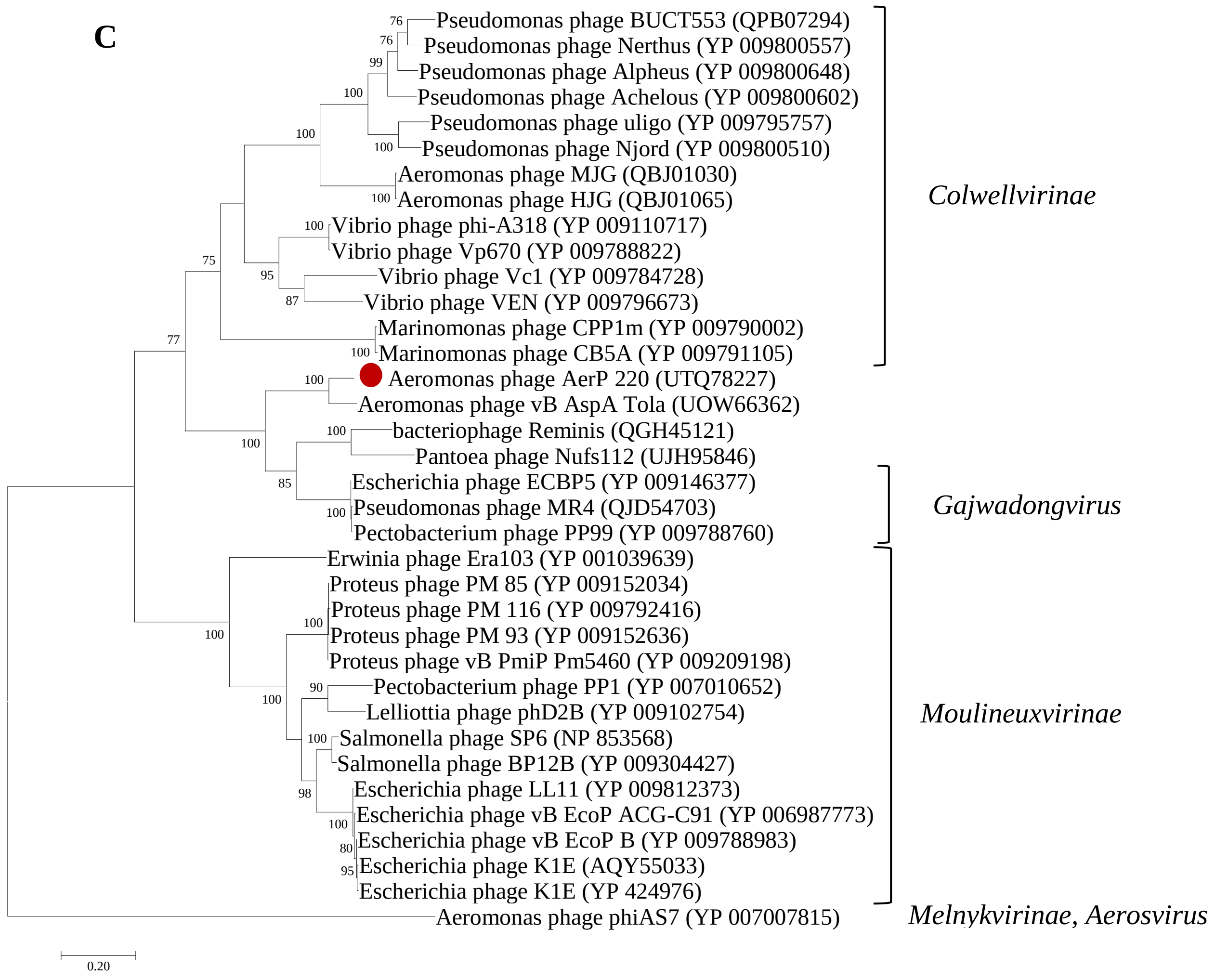

Figure S2. Phylogenetic analysis of three essential proteins of the investigated *Aeromonas* phage AerP_220 along with the most similar proteins: A, terminase large subunit; B, capsid protein; C, DNA-dependent RNA polymerase. GenBank identifiers for the protein sequences are shown in parentheses. The Maximum Likelihood method was used to construct the trees, 1000 bootstrap replications were applied. Statistical support above 70% is shown at the nodes. The sequence IDs of proteins of the investigated *Aeromonas* phage AerP_220 are marked with red circles. The appropriate proteins of *Aeromonas* phage phiAS7 were used as outgroup.
