# Supplementary material for: A Novel Aeromonas popoffii Phage AerP_220 Proposed to Be a Member of a New Tolavirus Genus in the Autographiviridae Family
Source: Viruses. 2022 Dec 7;14(12):2733. doi: 10.3390/v14122733 (PMC9780818; doi:10.3390/v14122733)
Supplement: Supplementary file 1 [file viruses-14-02733-s001.zip › Table S1.pdf]

Table S1. *Aeromonas* spp. strains screened in host range assay

No	Species name	CEMTC No	Growth temperature, °C	Isolation source
1	*A. allosaccharophila*	2090	30	river, Dalat, Vietnam
2	*A. caviae*	2395	37	open sewer drains, Phuket, Thailand
3	*A. hydrophila*	2089	30	river, Dalat, Vietnam
4	*A. hydrophila*	2094	30	river, Dalat, Vietnam
5	*A. jandaei*	1354	30	lake Bolshoe Yarovoe, Altay region, Russia
6	*A. jandaei*	1458	37	the Dudhsagar Waterfall, Goa, India
7	*A. media*	4531	25	pond, Novosibirsk, Russia
8	*A. popoffii*	1430	30	river Geysernaya, Death Valley, Kamchatka peninsula, Russia
9	*A. popoffii*	3381	22	lake Geysernoe , Ulagan district, Altai republic, Russia
10	***A. popoffii****	**4062**	28	river Inya, Novosibirsk region, Russia
11	*A. salmonicida*	1370	30	lake Bolshoe Yarovoe, Altay region, Russia
12	*A. salmonicida*	3364	30	lake Geysernoe, Altai republic, Russia
13	*A. salmonicida*	3375	30	lake Geysernoe, Altai republic, Russia
14	*A. salmonicida*	3383	22	lake Geysernoe, Altai republic, Russia
15	*A. salmonicida*	4051	25	stream at the foot of the Kizimen volcano, Kamchatka peninsula, Russia
16	*A. salmonicida*	4537	25	lake at the settlement Koinikha, Novosibirsk region, Russia
17	*A. salmonicida*	4546	25	mineral spring, Sakhalin island, Russia
18	*A. salmonicida/A. piscicola*	4331	25	lake Uryupino, Novosibirsk region, Russia
19	*A. sanarellii*	2912	37	cat dermatitis, veterinary clinics, Novosibirsk, Russia
20	*A. sobria*	4573	25	lake Malinovskoye, Novosibirsk region, Russia
21	*Aeromonas* sp.	4529	25	pond, Novosibirsk, Russia
22	*A. taiwanensis/A. dhakensis*	1617	30	Gulf of Thailand, Pattaya, Thailand
23	*A. veronii*	1362	30	lake Bolshoe Yarovoe, Altay region, Russia
24	*A. veronii*	1369	30	lake Bolshoe Yarovoe, Altay region, Russia
25	*A. veronii*	1414	30	thermal spring, Uzon Caldera, Kamchatka peninsula, Russia
26	*A. veronii*	1416	30	thermal spring, Uzon Caldera, Kamchatka peninsula, Russia
27	*A. veronii*	4064	28	river Inya, Novosibirsk region, Russia

*Host strain is marked with bold
