# Supplementary figures and images for: A Novel Aeromonas popoffii Phage AerP_220 Proposed to Be a Member of a New Tolavirus Genus in the Autographiviridae Family
Source: Viruses. 2022 Dec 7;14(12):2733. doi: 10.3390/v14122733 (PMC9780818; doi:10.3390/v14122733)

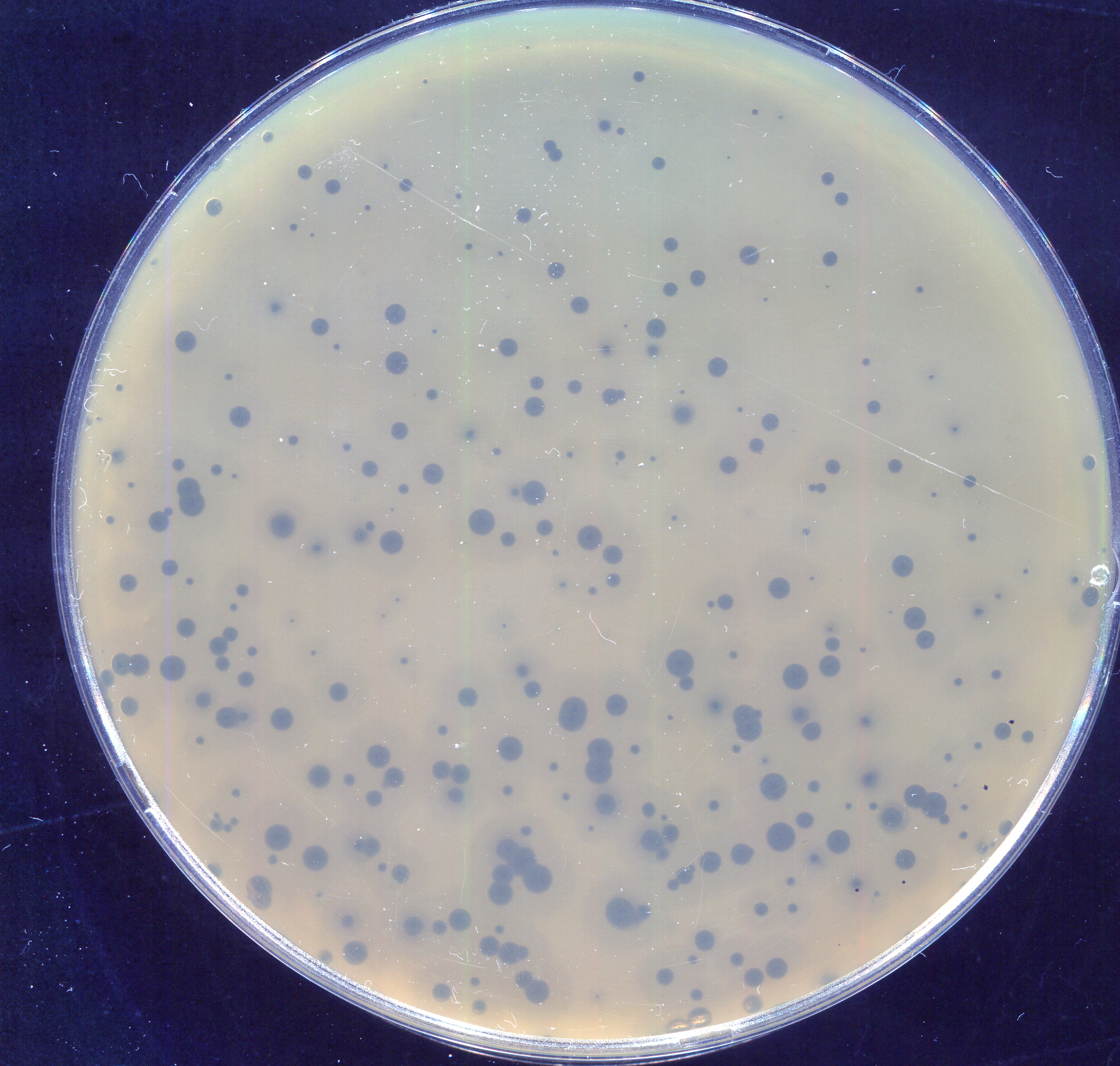

Supplement: Supplementary file 1 [file viruses-14-02733-s001.zip › Figure S1.jpg]
